# The long-term effects of tissue flossing on ankle range of motion, strength, balance, and jump performance in athletes with limited ankle dorsiflexion: a randomized controlled trial

**DOI:** 10.1186/s13102-026-01609-9

**Published:** 2026-02-24

**Authors:** Hassan Daneshmandi, Mohammad Alimoradi, Mohammad Alghosi, Omid Monfaredian, Amirhossein Barati, Urs Granacher

**Affiliations:** 1 Department of Sports Injury and Corrective Exercise, Faculty of Physical Education and Sport Sciences, University of Guilan, Rasht, Iran; 2 Department of Sports Injuries and Corrective Exercises, Faculty of Sports Sciences, Shahid Bahonar University of Kerman, Kerman, Iran; 3 HERC – Health, Exercise & Research Center, Mina Rashid, Dubai Maritime City, Dubai, United Arab Emirates; 4 Department of Physical Education, Technical and Vocational University (TVU), Tehran, Iran; 5 Department of Sports Biomechanics, Faculty of Sports Sciences, Shahid Bahonar University of Kerman, Kerman, Iran; 6 Department of Sports Medicine and Health, Faculty of Physical Education, University of Shahid Behshti, Tehran, Iran; 7 Department of Sport and Sport Science, Exercise and Human Movement Science, University of Freiburg, Freiburg, Germany

**Keywords:** Ankle joint, Floss band, Compression, Range of motion, Athletic performance

## Abstract

**Background:**

The long-term effects of tissue flossing (TF) on mobility, strength, balance, and muscle power remain unclear, particularly regarding its potential to produce performance enhancements. Here, we aimed to investigate the effects of TF on ankle range of motion (ROM), strength, balance, and jump performance in team- and racket-sport athletes with limited ankle dorsiflexion (DF).

**Methods:**

Forty male athletes (DF-ROM < 10°) were randomized into a TF group (*n* = 20) or a control group performing static stretching (SS, *n* = 20). The TF intervention employed a standard figure-of-eight bandaging technique with 50–70% overlap, consisting of active movements with elastic band compression, three times a week for six weeks, followed by a six-week detraining period. The SS group performed 3 × 30 s stretches for the gastrocnemius and soleus muscles. Outcomes included DF and plantarflexion (PF) ROM, isokinetic strength at 30°/s and 120°/s, Y-balance test (YBT), and Sargent jump test (SJT), measured at baseline, post-intervention, and post-detraining.

**Results:**

TF produced larger gains than SS in DF-ROM (*p* < 0.001, *d* = 4.52) and PF-ROM (*p* < 0.001, *d* = 0.90), with partial retention after detraining (DF: *p* < 0.001, *d* = 1.26; PF: *p* = 0.004, *d* = 0.22). Strength improved mainly in DF (*p* < 0.001, *d* = 0.36–0.78) and in PF at slower speeds (*p* < 0.001, *d* = 017-0.29), with partial retention (DF at 30 and 120°/s: *p* = 0.004–0.04, *d* = 0.04–0.13; PF at 30°/s: *p* = 0.04, *d* = 0.06). YBT scores increased in the posteromedial (*p* < 0.001, *d* = 0.84) and posterolateral (*p* = 0.001, *d* = 0.84) directions, but not anteriorly (*p* > 0.05). No significant effects were observed for SJT (*p* > 0.05).

**Conclusion:**

A six-week TF program effectively improved ankle ROM, strength, and dynamic balance in athletes with restricted DF, with many benefits persisting after detraining. Compared with static stretching, tissue flossing was more effective for long-term ankle function but did not enhance vertical jump performance. These results suggest that tissue flossing is a practical and efficient strategy for team- and racket-sport athletes seeking to restore and maintain ankle mobility and stability during training and rehabilitation.

**Trial registration:**

This trial was registered at the Iranian Registry of Clinical Trials (Identifier: IRCT20230612058457N7) on June 14, 2025.

**Supplementary Information:**

The online version contains supplementary material available at 10.1186/s13102-026-01609-9.

## Background

Limited ankle dorsiflexion (DF) is a common biomechanical issue affecting athletes across many sports [1]. DF is critical for athletic movements such as running, jumping, changing directions, and maintaining balance [2]. When ankle mobility is restricted, athletes may adopt compensatory movement patterns, resulting in reduced force efficiency, impaired stability, and an increased risk of injuries, including ankle sprains, Achilles tendinopathy, and patellar tendinopathy. These risks are particularly relevant during high-demand activities like squatting, cutting, and landing [3–5]. Among elite athletes, up to approximately 30% may experience clinically significant DF restrictions during the season, and up to 65% may exhibit signs of chronic ankle instability, conditions often associated with restricted ankle mobility [6, 7]. Traditional interventions to improve DF, such as stretching, soft tissue mobilization, and manual therapy, often require prolonged commitment and may yield inconsistent results [8–10]. Recently, tissue flossing (TF) has gained attention as a novel technique to enhance joint range of motion (ROM) [6] and athletic performance [11]. This method involves applying an elastic band around a joint or muscle group to create compression while performing active or passive movement [11]. The technique gained popularity following the publication by Starrett and Cordoza [12], who suggested that its effects are driven by fascial shearing and temporary blood flow restriction to the muscle. Additionally, the combination of compression and movement during flossing is thought to influence the interaction between the fascia and the neuromusculoskeletal system, enabling the fascia to move and stretch more freely. The combination of compression and movement is thought to temporarily restrict blood flow, alter fascial tension, and stimulate mechanoreceptors. Upon band removal, a reperfusion effect may enhance tissue pliability, ROM, and neuromuscular responsiveness [13, 14]. Although researchers have demonstrated short-term improvements in ROM and function following TF, its long-term effects remain largely unexplored [13]. In sports where agility, strength, and power are essential, sustained mobility improvements could translate into significant performance benefits [15, 16]. Emerging evidence suggests that interventions applying external compression can induce meaningful neuromuscular and sensorimotor adaptations that persist beyond the acute treatment phase, contributing to performance enhancement [17, 18]. These adaptations may include improved proprioceptive acuity, optimized motor unit recruitment, and improved movement efficiency, which are critical for long-term athletic performance and injury resilience. However, it is unclear whether regular use of TF can produce lasting gains in mobility, strength, balance, or muscle power. This study seeks to address that gap by examining the long-term effects (6 weeks) of TF on DF, muscular strength, balance, and jump performance in team- and racket-sport athletes with limited ankle mobility. The findings will inform whether TF is a viable, long-term strategy for improving key physical attributes in sport. Additionally, we hypothesize that any improvements in DF, strength, balance, and jump performance achieved through TF will be sustained even after a 6-week detraining period, providing insight into the potential long-term benefits of this intervention for athletic performance.

## Methods

### Study Design and Setting

This randomized controlled trial was conducted from June 24, 2025, to October 2, 2025, to evaluate the long-term effects of TF on ankle ROM, muscular strength, balance, and jump performance in athletes with limited ankle mobility. The trial was conducted over a twelve-week period at a university-affiliated sports performance laboratory and athletic training center, which included six weeks of intervention followed by six weeks of detraining. The facility was equipped with standardized testing tools for biomechanical, neuromuscular, and performance assessment, ensuring consistency in data collection. Participants were recruited from collegiate and semi-professional sport teams in the region, including soccer (*n* = 19), basketball (*n* = 10), badminton (*n* = 8) and tennis (*n* = 7). Based on the McKay et al. [19] classification, this cohort comprised Tier 3 athletes, defined as highly trained individuals who competed in structured leagues and participated in supervised training for over 8 h weekly. The study was approved by the Institutional Review Board of Guilan University (IR.GUILAN.REC.1404.017) on April 14, 2025. All participants attended a 2-hour familiarization session 48 h before the baseline assessment to learn about the tests and complete consent and personal information forms. Written informed consent was obtained from all participants prior to their participation. Randomization was performed using www.randomizer.org website by one of the researchers in a 1:1 ratio to allocate participants into two groups: an intervention group receiving tissue flossing (TF; *n* = 22) and an active control group performing static stretching (SS; *n* = 22). The study was registered with a clinical trial registry (IRCT20230612058457N7) on June 14, 2025. The authors followed the principles of the Declaration of Helsinki.

### Participants

**Fig. 1:**
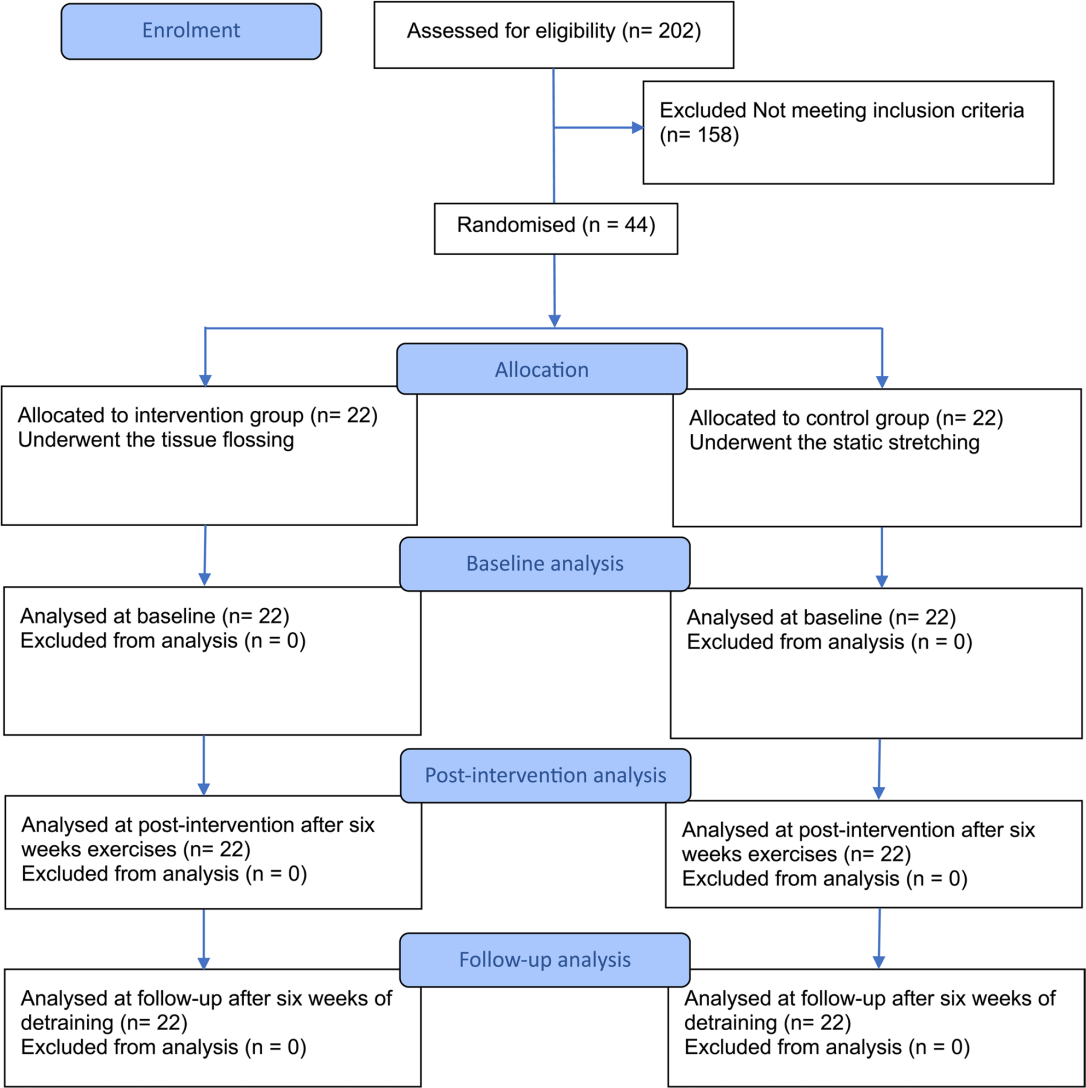
Flowchart illustrating the progression of participants through enrollment, allocation, intervention, and analysis stages

From 202 initially screened male athletes, 44 were enrolled in the study (mean age: 25.9 ± 4.4 years; height: 179.1 ± 3.3 cm; mass: 75.5 ± 3.1 kg). Participants were aged 18–35 years and actively engaged in competitive team- and racket-sports. Inclusion criteria required participants to have a DF-ROM of less than 10º, assessed via the weight-bearing lunge test using a goniometer (as detailed in the Measurements section), and no significant lower limb injury within the past six months. All individuals were expected to adhere to the intervention protocol and attend scheduled sessions consistently. Exclusion criteria included the use of an ankle brace during the study period, absence from two consecutive sessions or three non-consecutive sessions, unwillingness to continue participation at any point during the study, and the use of performance-enhancing therapies or supplements that could affect mobility or strength. All eligible participants provided written informed consent. Figure 1 presents the study progression, detailing participant enrollment, allocation, and analysis.

### Procedures

**Fig. 2:**
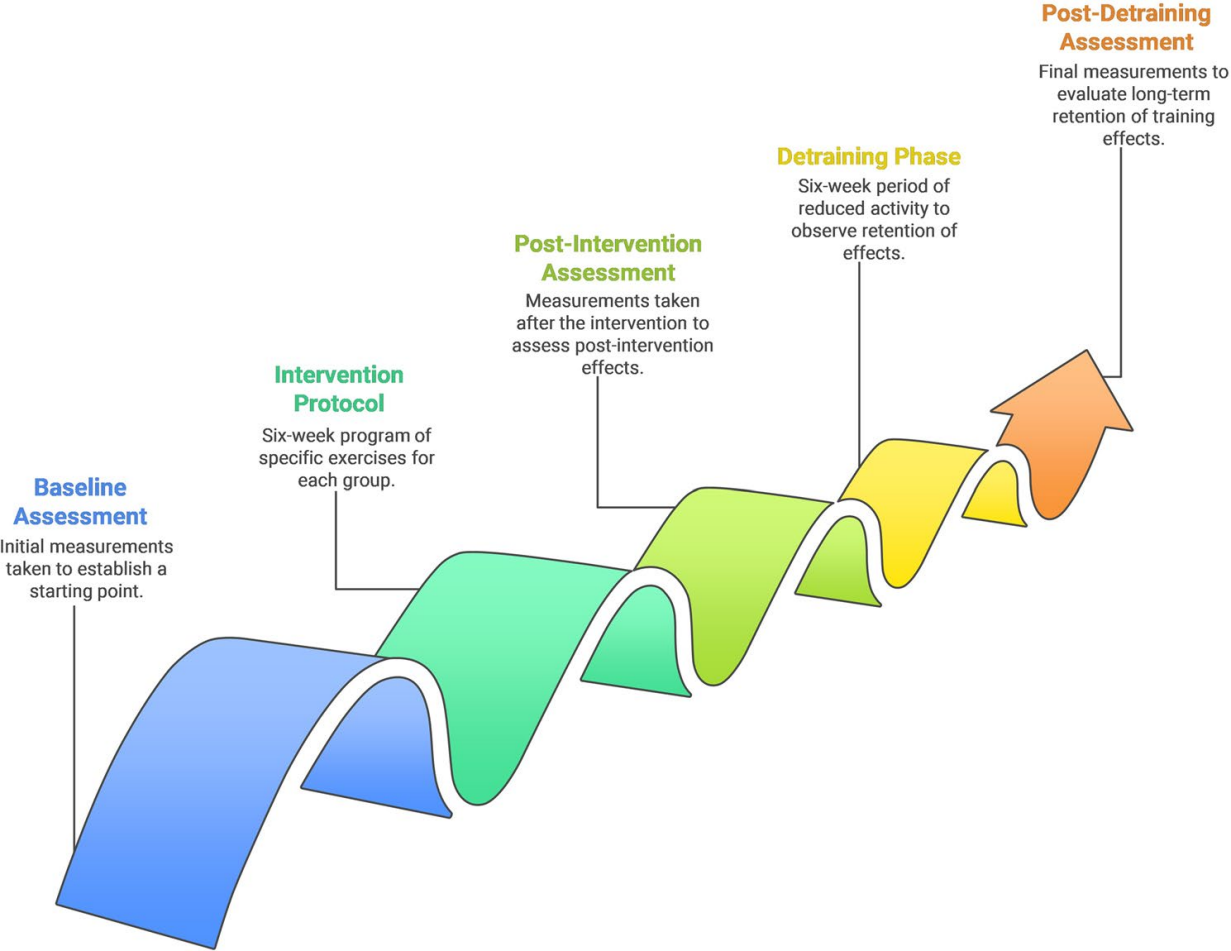
Study timeline and intervention flow. The six-week tissue flossing or static stretching intervention was conducted three times per week, followed by a six-week detraining period. Assessments were performed at baseline, post-intervention (week 6), and post-detraining (week 12)

**Fig. 3:**
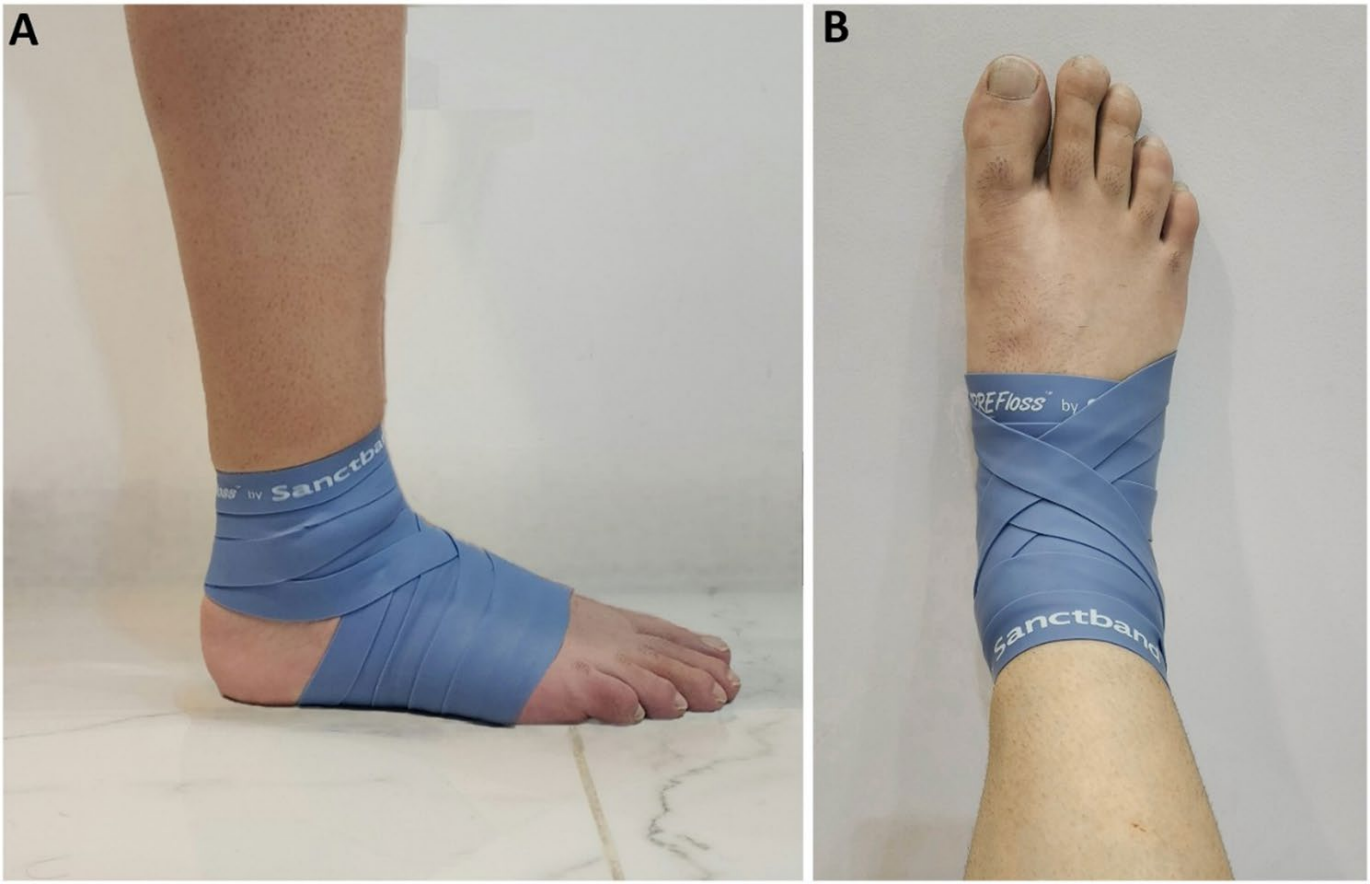
Application of the floss band to the ankle using a standard figure-of-eight wrapping technique. (**A**) Lateral view: the band is wrapped with 50–70% overlap around the ankle joint, covering the lateral malleolus, Achilles tendon, and medial malleolus. (**B**) Anterior view: the wrap extends across the dorsum of the foot to secure compression during active movement exercises

All participants completed a six-week intervention protocol followed by a six-week detraining phase to evaluate the retention of training effects. Assessments were conducted at three time points: baseline, post-intervention (Week 6), and post-detraining (Week 12) (Fig. 2). All participants began each intervention session with a five-minute bike warm-up. For participants in the TF group, an elastic floss band (Sanctband COMPRE Floss, Blueberry; PENTEL SDN.BHD., Shah Alam, Malaysia), sized 2 m × 5 cm, was wrapped around the ankle joint using a standard technique. This involved two circular wraps around the foot’s transverse arch followed by three figure-of-eight wraps (from lateral malleolus, around the Achilles tendon, to medial malleolus, and back), with each subsequent wrap overlapping the previous by approximately 50–70% (Fig. 3) [2, 20]. While the band was applied, participants performed active movements including ankle pumps, bodyweight squats, and lunges. Each exercise was performed in three sets with protocol-specific durations, and the entire intervention was repeated three times per week over six weeks. Moreover, interface pressure between the skin and the floss band was measured to assess the level of compression (in mmHg) achieved by the wrapping technique. A Kikuhime pressure monitor sensor (MediGroup, Melbourne, Australia) was placed on the anterior aspect of the tibia at the midline between the lateral and medial malleolus during wrapping to ensure consistent and safe pressure application This device demonstrates an intraclass coefficient (ICC) of 0.99, indicating excellent reliability for use in sports medicine settings [21]. Participants in the SS group performed active stretching exercises designed to improve ankle mobility. These exercises targeted the calf muscles (gastrocnemius and soleus), with each stretch held for 30 s, repeated for three sets with 15 s of rest between sets. This stretching routine was performed three times weekly for six weeks. Both groups completed their interventions at the same time of day (± 1 h) to minimize circadian variation, and all sessions were supervised by trained staff to ensure proper execution. No adverse events or complications were reported during the intervention period. During the six-week detraining phase, participants refrained from any ankle-specific mobility or strength exercises, though they were permitted to continue general athletic training without additional recovery modalities. Testing followed a fixed sequence: (1) DF and PF-ROM, (2) DF and PF strength, (3) Y-Balance Test (YBT), and (4) Sargent jump test (SJT), with a 5-minute rest between tests and a 2-minute rest between trials. All measurements were performed on the dominant leg, with dominance defined as the preferred kicking leg [22]. Moreover, no adverse events occurred in either of the studied groups. ICCs were calculated for all outcome variables using test-retest data collected 48 h apart during familiarization and baseline assessments, with ICC > 0.80 considered indicative of good reliability. All outcome measures demonstrated excellent reliability (ICC range: 0.92–0.99). Standard error of measurement, minimal detectable change values and coefficient of variation were also computed. Full intervention protocols for both groups, including exercise duration and set/rep schemes, are provided in Supplementary File 1. Reliability statistics are presented in Supplementary File 2.

### Measurements

#### DorsiFlexion and PlantarFlexion-Range Of Motion

DF-ROM was measured using a goniometer while the participant stood in a weight-bearing position facing a wall. The big toe of the front foot was positioned 25 cm from the wall, with the knee aligned with the second toe and the heel in contact with the ground. To maintain balance, participants were allowed to lightly touch the wall with two fingers from each hand. They were then instructed to move their knee straight toward the wall without lifting the heel, continuing until the knee touched the wall. The foot was advanced toward the wall in 1 cm increments until the participant could touch the wall with the knee without the heel lifting. At that point, the measurement was taken using the goniometer (Ghamatpooyan, Tehran, Iran). The center of the goniometer was placed precisely over the lateral malleolus, with one arm aligned parallel to the fibula and the other parallel to the fifth metatarsal bone [23]. To measure PF-ROM, Participants were positioned barefoot lying on their back with their knees flexed between 25–30° to minimize tension in the gastrocnemius muscle. The goniometer’s axis was aligned near the lateral malleolus, while its fixed arm was placed parallel to the lateral aspect of the fibula, and the movable arm was aligned with the lateral surface of the fifth metatarsal. Participants then actively moved their foot into PF, and the angle achieved was recorded from the neutral position [23]. These measurements were performed three times, and the average of the three trials was recorded as the final value.

#### Dorsiflexion and Plantarflexion Muscle Strength

Isokinetic dynamometry (Biodex Medical Systems, Shirley, NY, USA) of the ankle dorsiflexor and plantarflexor groups was performed to assess peak torque. Positioning orientations were according to the manufacturer’s specifications: supine position with the chair inclination at 25; thorax, abdomen, and distal third-thigh cross straps; dynamometer perpendicular to the chair and parallel to the floor; and ankle initially positioned in neutral position, keeping the tibia parallel to the floor and the foot fixed at 90, forming a right angle. Testing was performed at 2 angular speeds, 30 (slow) and 120 deg/s (fast), in the concentric (CON) and eccentric (ECC) modes, with three maximal repetitions at each speed and 60 s of recovery between tests [24]. The average of the trials was recorded as the final value. During the test, the participant was asked to perform the maximal voluntary isometric contraction, with verbal stimulation during the execution of the movement.

#### Dynamic Test

Participants performed the YBT barefoot, standing on one dominant leg while reaching as far as possible with the contralateral leg in three directions: anterior, posteromedial, and posterolateral. Each direction was tested three times with a passive recovery period of 10 s between them, and the maximum reach distance (in centimeters) was recorded [25, 26]. The order of testing directions was randomized to minimize learning effects. The mean and standard deviation for each direction were determined by using the range of values obtained from the test along with the actual length of the limbs measured for value normalization. To facilitate comparison between athletes, the range measurement was adjusted for limb length. The normalized value was determined by dividing the total of the three range values by three times the length of the limb and then multiplying it by 100 (Eq. 1) [22].

1 $$\mathrm{YBT}_{Normalized}=\left(\frac{\mathrm{Anterior}+\mathrm{Posteromedial}+\mathrm{Posterolateral}}{3\:\times\:\mathrm{Limb}\:\mathrm{length}}\right)\times\:100$$

#### Sargent Jump Test 

During SJT performance, participants stand, beside a vertical jump board or wall. First, their standing reach height is measured. Then, they perform a vertical jump, attempting to touch the board or wall at the peak of their jump. A sensor system (Ghamatpooyan, Tehran, Iran) records the difference between the standing reach height and the jump height, providing a measurement of the vertical jump distance [27]. This test is commonly used in sports to evaluate lower limb strength and power. Each participant performs three jumps, with a minimum of 45 s of rest between attempts. Only the highest jump is recorded [27].

### Data Analyses

The a priori sample size was calculated based on findings from a related study on the effects of tissue flossing on DF- ROM in athletes with chronic ankle instability [28] using the G*Power software (version 3.1.9.4; University of Kiel, Kiel, Germany). As input parameters, a Cohen’s *f* effect size of *f* = 0.28 was used for the primary outcome, DF ROM. In addition, an alpha error of *p* < 0.01 was included (5% probability of type I error), and β = 0.90 (90% statistical power), a minimum of 40 participants was required. To accommodate an estimated 10% attrition rate, the target sample size was increased to 44 participants.

Statistical analyses were conducted using SPSS (version 27.0; IBM Corp., Armonk, NY, USA.). The normality of data distribution was confirmed using the Shapiro-Wilk test, with a significance threshold of *p* > 0.05. A 2 × 3 repeated-measures analysis of variance (ANOVA) was employed to examine the main effects of time (baseline, post-intervention, follow-up) and group (TF, SS), as well as their interaction (group-by-time). In cases where a significant interaction effect was found, post-hoc pairwise comparisons were carried out using t-tests adjusted with a Bonferroni correction. For all significant results, effect sizes were calculated. Partial eta squared (η²) was used for the ANOVA results and was subsequently converted to Cohen’s *d* for interpretation. Based on established guidelines [29], within-group Cohen’s *d* values were interpreted as small (0.00–0.49), moderate (0.50–0.79), or large (≥ 0.80). The alpha level for statistical significance was set at *p* < 0.05 for all tests, which used a 95% confidence interval.

## Results

Out of the 44 athletes who were enrolled and randomized, 40 completed the study and were included in the final analysis (Fig. 1). Two participants from the SS group withdrew during the second week due to personal reasons. Additionally, two participants from the TF group did not participate in the post-detraining assessment. The baseline characteristics of the participants who completed the study are presented in Table 1. There were no significant differences in anthropometric characteristics among the groups at baseline (*p* > 0.05).

**Table 1:** Participant demographics by group

Variables	TF Mean ± SD	SS Mean ± SD	*p*-value
Age (year)	26.6 ± 4.4	25.2 ± 4.7	0.36
Body height (cm)	179.5 ± 3.1	178.1 ± 3.4	0.18
Body mass (kg)	75.8 ± 3.1	74.2 ± 2.5	0.08
Body mass index (kg/m^2^)	23.5 ± 1.4	23.4 ± 1.4	0.81
Sport-specific training experience (year)^¥^	7.2 ± 2.2	6.1 ± 2.7	0.16
Leg dominance (N)	18 R/ 2 L	19 R/ 1 L	
Limb length (cm) ^*^	83.4 ± 1.6	82.6 ± 1.3	0.13
Baseline DF (°)^ǂ^	7.5 ± 1.1	8.1 ± 0.8	0.06

Abbreviation: *DF *dorsiflexion, *SD *standard deviation, *TF *tissue flossing, *SS *static stretching

Significant level set as *p* < 0.05.  ^*^Length of dominant leg was assessed for Y balance test normalization; ^ǂ^Assessed in familiarization session

^¥^Refers to the number of years the participant has been actively training and competing

### Dorsiflexion Range of Motion

Statistical analysis indicated significant effects for both time (F = 175.02, *p* < 0.001, *d* = 0.82; large) and group-by-time interaction (F = 36.71, *p* < 0.001, *d* = 0.49; small). In addition, a significant group effect was observed (F = 6.65, *p* = 0.014, *d* = 0.14; small). Post-hoc comparisons demonstrated that the TF group exhibited significantly elevated scores at post-intervention relative to both baseline (*p* < 0.001, *d* = 4.52; large, 95% CI 4.09 to 5.92) and post-detraining assessments (*p* < 0.001, *d* = 3.25; large, 95% CI 2.87 to 4.25), with post-detraining values also exceeding baseline levels (*p* < 0.001, *d* = 1.26; large, 95% CI 0.96 to 1.92). Similarly, the SS group showed significantly higher post-intervention scores compared to both baseline (*p* < 0.001, *d* = 2.50; large, 95% CI 1.63 to 2.28) and post-detraining measurements (*p* < 0.001, *d* = 1.55; large, 95% CI 0.75 to 1.39), while maintaining higher post-detraining scores than baseline (*p* < 0.001, *d* = 1.27; large, 95% CI 0.52 to 1.23). Additionally, a significant difference between the TF and SS groups was observed at post-intervention (*p* < 0.001, *d* = 2.52; large, 95% CI 1.80 to 3.03) (Table 2).

**Table 2:** Comparisons of the outcomes (Mean ± SD) among the studied groups at baseline, post-intervention and post-detraining

Variable	TF			SS		
	Baseline	Post	Detraining	Baseline	Post	Detraining
DF-ROM (**°**)	7.5 ± 1.2	12.5 ± 1.0^*,**^ ↑66.66%^Ψ^-↑40.44%^ǂ^	8.9 ± 1.0^***^ ↑18.66%^¥^	8.1 ± 0.8	10.1 ± 0.8^*,**^ ↑24.69% ^Ψ^ -↑12.22%^ǂ^	9.0 ± 0.6^***^ ↑11.11%^¥^
PF-ROM (**°)**	41.1 ± 5.3	45.7 ± 4.9^*,**^ ↑11.19% ^Ψ^ -↑8.29%^ǂ^	42.2 ± 4.6^***^ ↑2.67%^¥^	41.5 ± 2.6	43.0 ± 2.7^*,**^ ↑3.61% ^Ψ^ -↑2.38%^ǂ^	42.0 ± 2.3^***^ ↑1.20%^¥^
**Isokinetic strength-30°/s**						
DF-CON (Nm)	50.1 ± 12.5	54.4 ± 11.2^*,**^ ↑8.58% ^Ψ^ -↑5.22%^ǂ^	51.7 ± 12.1^***^ ↑3.19%^¥^	50.6 ± 11.5	51.3 ± 11.3 ↑1.38% ^Ψ^ -↑1.18%^ǂ^	50.7 ± 11.5 ↑0.19%^¥^
DF-ECC (Nm)	74.3 ± 12.2	81.8 ± 10.6^*,**^ ↑10.09% ^Ψ^ -↑8.63%^ǂ^	75.3 ± 12.2^***^ ↑1.34%^¥^	78.1 ± 13.4	78.8 ± 13.3^*^ ↑0.89% ^Ψ^ -↑0.25%^ǂ^	78.6 ± 13.4^***^ ↑0.64%^¥^
PF-CON (Nm)	164.7 ± 23.8	171.4 ± 21.9^*,**^ ↑4.06% ^Ψ^ -↑3.12%^ǂ^	166.2 ± 23.1^***^ ↑0.91%^¥^	163.5 ± 20.7	165.3 ± 19.9^*,**^ ↑1.10% ^Ψ^ ↑-0.66%^ǂ^	164.2 ± 20.2^***^ ↑0.42%^¥^
PF-ECC (Nm)	224.9 ± 51.3	233.9 ± 52.3^*,**^ ↑4% ^Ψ^ -↑3.13%^ǂ^	226.8 ± 51.1 ↑0.84%^¥^	233.0 ± 39.5	233.6 ± 39.2^*,**^ ↑0.25% ^Ψ^ -↑0.21%^ǂ^	233.1 ± 39.5 ↑0.04%^¥^
**Isokinetic strength-120°/s**						
DF-CON (Nm)	35.1 ± 6.0	40.0 ± 6.5^*,**^ ↑13.96% ^Ψ^ -↑12.04%^ǂ^	35.7 ± 6.1^***^ ↑1.70%^¥^	37.2 ± 7.4	38.2 ± 7.5^*,**^ ↑2.68% ^Ψ^ -↑1.32%^ǂ^	37.7 ± 7.5^***^ ↑1.34%^¥^
DF-ECC (Nm)	75.1 ± 13.3	80.0 ± 11.7^*,**^ ↑6.52% ^Ψ^ -↑5.68%^ǂ^	75.7 ± 13.0^***^ ↑0.79%^¥^	77.3 ± 12.8	77.8 ± 12.5^*,**^ ↑0.64% ^Ψ^ -↑0.38%^ǂ^	77.5 ± 12.8 ↑0.25%^¥^
PF-CON (Nm)	106.0 ± 18.0	107.2 ± 16.1 ↑1.13% ^Ψ^ -↑0.18%^ǂ^	107.0 ± 17.7 ↑0.94%^¥^	113.8 ± 19.8	113.9 ± 19.5 ↑0.08% ^Ψ^ -↑0.26%^ǂ^	113.6 ± 18.9 ↓0.17%^¥^
PF-ECC(Nm)	220.3 ± 47.8	230.7 ± 40.6 ↑4.72% ^Ψ^ -↑2.80%^ǂ^	224.4 ± 40.7 ↑1.86%^¥^	229.9 ± 46.7	229.7 ± 46.3 ↓0.08% ^Ψ^ -↑0.61%^ǂ^	228.3 ± 44.8 ↓0.69%^¥^
**Normalized Y-balance-test**						
YBT-ANT (cm)	99.6 ± 10.7	103.1 ± 4.5 ↑3.51% ^Ψ^ -↑3.41%^ǂ^	99.7 ± 5.9 ↑0.10%^¥^	100.3 ± 10.0	101.5 ± 5.3 ↑1.19% ^Ψ^ -↑1.39%^ǂ^	100.1 ± 8.8 ↓0.19%^¥^
YBT-ML (cm)	97.4 ± 11.1	107.5 ± 12.7^*,**^ ↑10.36% ^Ψ^ -↑6.12%^ǂ^	101.3 ± 8.2^***^ ↑4%^¥^	100.4 ± 6.9	98.2 ± 4.2^*,**^ ↓2.19% ^Ψ^ -↓1.80%^ǂ^	100.0 ± 5.7 ↓0.39%^¥^
YBT-PL (cm)	104.0 ± 9.1	111.8 ± 9.4^*^ ↑7.50% ^Ψ^ -↑2.85%^ǂ^	108.7 ± 11.1^***^ ↑4.51%^¥^	102.3 ± 7.4	103.8 ± 7.5 ↑1.46% ^Ψ^ -↓0.66%^ǂ^	104.5 ± 7.7 ↑2.15%^¥^
Total score (cm)	100.3 ± 6.8	107.5 ± 5.7^*,**^ ↑7.17% ^Ψ^ -↑4.16%^ǂ^	103.2 ± 5.8^***^ ↑2.89%^¥^	101.0 ± 5.6	101.2 ± 3.4 ↑0.19% ^Ψ^ -↓0.29%^ǂ^	101.5 ± 5.1 ↑0.49%^¥^
SJT (cm)	43.4 ± 3.0	44.0 ± 2.9 ↑1.38% ^Ψ^ -↑2.56%^ǂ^	42.9 ± 1.8 ↓1.15%^¥^	42.4 ± 3.1	41.9 ± 2.2 ↓1.17% ^Ψ^ -↑0.47%^ǂ^	41.7 ± 2.0 ↓1.65%^¥^

Abbreviations: *DF *dorsiflexion, *ROM *range of motion, *PF *plantarflexion, *CON *concentric, *ECC *eccentric, *YBT *Y balance test, *ANT *anterior, *PM *posteromedial, *PL *posterolateral, *SJT *Sargent jump test, *TF *tissue flossing, *SS *static stretching

^*^ Significant difference between post-intervention and baseline ^**^Significant difference between post-intervention and post-detraining ^***^ Significant difference between post-detraining and baseline^Ψ^ Percentage change from post-intervention to baseline; ^ǂ^Percentage change from post-intervention to post-detraining; ^¥^Percentage change from post-detraining to baseline

### Plantarflexion Range of Motion

The analysis revealed a significant main effect of time (F = 68.92, *p* < 0.001, *d* = 0.64; moderate) and a significant group-by-time interaction (F = 18.15, *p* < 0.001, *d* = 0.32; small), but no significant group effect (F = 0.45, *p* = 0.50, η^2^ = 0.01; small). Post-hoc analysis showed that in the TF group, post-intervention values were significantly higher than both baseline (*p* < 0.001, *d* = 0.90; large, 95% CI 3.38 to 5.85) and post-detraining values (*p* < 0.001, *d* = 0.73; moderate, 95% CI 4.65 to 6.36), with post-detraining values also being significantly higher than baseline (*p* = 0.004, *d* = 0.22; small, 95% CI 0.39 to 1.83). Similarly, in the SS group, post-intervention values were significantly higher than both baseline (*p* < 0.001, *d* = 0.56; moderate, 95% CI 1.15 to 1.90) and post-detraining values (*p* < 0.001, *d* = 0.39; small, 95% CI 0.54 to 1.50), while post-detraining values remained significantly higher than baseline (*p* = 0.006, *d* = 0.20; small, 95% CI 0.16 to 0.84) (Table 2).

### Dorsiflexion Strength at 30 and 120°/s

At 30°/s in CON mode, the analysis demonstrated significant effects for both time (F = 93.49, *p* < 0.001, *d* = 0.71; moderate) and group-by-time interaction (F = 70.68, *p* < 0.001, *d* = 0.65; moderate), while no significant group effect was observed (F = 0.12, *p* = 0.72, *d* = 0.003; small). Post-hoc analysis demonstrated that the TF group showed significantly higher values at post-intervention compared to both baseline (*p* < 0.001, *d* = 0.36; small, 95% CI 3.48 to 5.12) and post-detraining (*p* < 0.001, *d* = 0.23; small, 95% CI 2.12 to 3.44), with post-detraining values also being significantly higher than baseline (*p* = 0.004, *d* = 0.13; small, 95% CI 1.00 to 2.03). In contrast, the SS group exhibited no significant differences across time points (*p* > 0.05) (Table 2). In ECC mode, there was a significant time effect (F = 66.27, *p* < 0.001, *d* = 0.63; moderate), a group-by-time interaction (F = 50.29, *p* < 0.001, *d* = 0.57; moderate), while no significant group effect was found (F = 0.11, *p* = 0.73, *d* = 0.003; small). Post-hoc analysis revealed significantly higher values at post-intervention compared to baseline (*p* < 0.001, *d* = 0.65; moderate, 95% CI 5.72 to 9.33) and post-detraining (*p* < 0.001, *d* = 0.56; moderate, 95% CI 4.60 to 8.54), as well as significantly higher values at post-detraining compared to baseline (*p* = 0.004, *d* = 0.08; small, 95% CI 0.45 to 1.45) in the TF group. Moreover, the SS group revealed significantly higher values at post-intervention compared to baseline (*p* < 0.001, *d* = 0.05; small, 95% CI 0.36 to 1.05) and post-detraining compared to baseline (*p* < 0.001, *d* = 0.03; small, 95% CI 0.30 to 0.77) (Table 2).

At 120°/s in CON mode, the analysis demonstrated a significant main effect of time (F = 162.91, *p* < 0.001, *d* = 0.81; large) as well as a significant group-by-time interaction (F = 81.22, *p* < 0.001, *d* = 0.68; moderate). However, there was no significant group effect (F = 0.12, *p* = 0.72, *d* = 0.003; small). Post-hoc analysis of the group-by-time interaction showed significantly higher values at post-intervention compared to baseline (*p* < 0.001, *d* = 0.78; moderate, 95% CI 4.03 to 5.70) and post-detraining (*p* < 0.001, *d* = 0.68; moderate, 95% CI 3.50 to 5.00), as well as significantly higher values at post-detraining compared to baseline (*p* = 0.004, *d* = 0.09; small, 95% CI 0.28 to 0.94) in the TF group. Moreover, the SS group revealed significantly higher values at post-intervention compared to baseline (*p* < 0.001, *d* = 0.13; small, 95% CI 0.67 to 1.32) and post-detraining (*p* < 0.001, *d* = 0.06; small, 95% CI 0.33 to 0.66), as well as significantly higher values at post-detraining compared to baseline (*p* < 0.001, *d* = 0.06; small, 95% CI 0.25 to 0.73) (Table 2). In ECC mode, the analysis showed a significant main effect of time (F = 37.57, *p* < 0.001, *d* = 0.49; small) as well as a significant group-by-time interaction (F = 27.63, *p* < 0.001, *d* = 0.42; small). However, there was no significant group effect (F = 0.02, *p* = 0.88, *d* = 0.001; small). Post-hoc analysis of the group-by-time interaction demonstrated significantly higher values at post-intervention compared to baseline (*p* < 0.001, *d* = 0.39; small, 95% CI 3.13 to 6.60) and post-detraining (*p* < 0.001, *d* = 0.34; small, 95% CI 2.80 to 5.90), as well as significantly higher values at post-detraining compared to baseline (*p* = 0.04, *d* = 0.04; small, 95% CI 0.01 to 1.01) in the TF group. Moreover, the SS group revealed significantly higher values at post-intervention compared to baseline (*p* = 0.002, *d* = 0.03; small, 95% CI 0.17 to 0.65) and post-detraining (*p* = 0.03, *d* = 0.02; small, 95% CI 0.02 to 0.51) (Table 2).

### Plantarflexion Strength at 30 and 120°/s

At 30°/s in CON mode, the analysis revealed a significant main effect of time (F = 66.83, *p* < 0.001, *d* = 0.63; moderate) as well as a significant group-by-time interaction (F = 23.95, *p* < 0.001, *d* = 0.38; small). However, there was no significant group effect (F = 0.20, *p* = 0.65, *d* = 0.005; small). Post-hoc analysis demonstrated significantly higher values at post-intervention compared to baseline (*p* < 0.001, *d* = 0.29; small, 95% CI 5.01 to 8.44) and post-detraining (*p* < 0.001, *d* = 0.23; small, 95% CI 3.66 to 6.85), as well as significantly higher values at post-detraining compared to baseline (*p* = 0.04, *d* = 0.06; small, 95% CI 0.79 to 2.15) in the TF group. Moreover, the SS group revealed significantly higher values at post-intervention compared to baseline (*p* = 0.001, *d* = 0.08; small, 95% CI 0.77 to 2.79) and post-detraining (*p* = 0.004, *d* = 0.05; small, 95% CI 0.39 to 1.85), as well as significantly higher values at post-detraining compared to baseline (*p* = 0.01, *d* = 0.03; small, 95% CI 0.17 to 1.15) (Table 2).In ECC mode, the analysis showed a significant main effect of time (F = 20.93, *p* < 0.001, *d* = 0.35; small) as well as a significant group-by-time interaction (F = 15.74, *p* < 0.001, *d* = 0.29; small). However, there was no significant group effect (F = 0.10, *p* = 0.74, η² = 0.003; small). Post-hoc analysis demonstrated significantly higher values at post-intervention compared to baseline (*p* < 0.001, *d* = 0.17; small, 95% CI 4.85 to 5.12) and post-detraining (*p* < 0.001, *d* = 0.13; small, 95% CI 3.71 to 10.40) in the TF group. Moreover, the SS group revealed significantly higher values at post-intervention compared to baseline (*p* = 0.006, *d* = 0.01; small, 95% CI 0.20 to 1.07) and post-detraining (*p* = 0.014, *d* = 0.002; small, 95% CI 0.11 to 0.89) (Table 2).

At 120°/s in CON mode, the analysis revealed no significant main effect of time (*F* = 1.66, *p* = 0.19, *d* = 0.04; small), no group-by-time interaction (*F* = 1.79, *p* = 0.17, *d* = 0.04; small), and no significant group effect (*F* = 1.45, *p* = 0.23, *d* = 0.03; small) (Table 2). In ECC mode, only the main effect of time was significant (*F* = 3.72, *p* = 0.029, *d* = 0.08; small), whereas the group-by-time interaction (*F* = 2.88, *p* = 0.062, *d* = 0.07; small) and the group effect (*F* = 0.07, *p* = 0.78, *d* = 0.002; small) were not significant (Table 2).

### Y-Balance Test

In anterior direction, the analysis showed no significant main effect of time (*F* = 1.33, *p* = 0.27, *d* = 0.03; small), no significant group-by-time interaction (*F* = 0.27, *p* = 0.76, η² = 0.007; small), and no significant group effect (*F* = 0.01, *p* = 0.91, *d* = 0.001; small) (Table 2). In posteromedial direction, the main effect of time (*F* = 7.54, *p* = 0.001, η² = 0.16; small) and group-by-time interaction (*F* = 19.26, *p* < 0.001, η² = 0.33; small) were significant. However, there was no significant group effect (F = 1.03, *p* = 0.31, η² = 0.02; small). Post-hoc analysis revealed significantly higher values at post-intervention compared to baseline (*p* < 0.001, *d* = 0.84; large, 95% CI 6.54 to 13.56) and post-detraining (*p* = 0.013, *d* = 0.62; moderate, 95% CI 1.48 to 10.84), as well as significantly higher values at post-detraining compared to baseline (*p* = 0.020, *d* = 0.44; moderate, 95% CI 0.67 to 7.10) in the TF group. In the SS group, values were significantly lower at post-intervention compared to baseline (*p* = 0.039, *d* = 0.38; small, 95% CI 0.12 to 4.38) and post-detraining (*p* = 0.014, *d* = 0.35; small, 95% CI 0.40 to 3.19) (Table 2). In posterolateral direction, there was a significant time effect (F = 8.84, *p* < 0.001, *d* = 0.18; small), a group-by-time interaction (F = 3.81, *p* = 0.005, *d* = 0.09; small). However, there was no significant group effect (F = 3.48, *p* = 0.07, η² = 0.08; small). Post-hoc analysis showed significantly higher values at post-intervention compared to baseline (*p* = 0.001, *d* = 0.84; large, 95% CI 3.43 to 12.22), and significantly higher values at post-detraining compared to baseline (*p* = 0.009, *d* = 0.46; moderate, 95% CI 1.31 to 8.04) in the TF group. No significant differences were found in the SS group (*p* > 0.05) (Table 2). For total score, the main effect of time (*F* = 11.80, *p* < 0.001, *d* = 0.23; small) and group-by-time interaction (*F* = 11.11, *p* < 0.001, *d* = 0.22; small) were significant. However, there was no significant group effect (F = 2.55, *p* = 0.11, *d* = 0.06; small). Post-hoc analysis revealed significantly higher values at post-intervention compared to baseline (*p* < 0.001, *d* = 1.14; large, 95% CI 4.64 to 9.60) and post-detraining (*p* < 0.001, *d* = 0.74; large, 95% CI 2.38 to 6.08). Additionally, values at post-detraining were significantly higher than at baseline (*p* = 0.028, *d* = 0.45; moderate, 95% CI 0.35 to 5.42) in the TF group. No significant differences were found in the SS group (*p* > 0.05) (Table 2).

### Sargent Jump Test

The analysis demonstrated no significant main effect of time (F = 1.92, *p* = 0.15, *d* = 0.04; small), no group-by-time interaction (F = 1.26, *p* = 0.28, *d* = 0.03; small), and no significant group effect (F = 4.10, *p* = 0.050, *d* = 0.09; small) (Table 2).

## Discussion

The present study investigated the long-term effects of a six-week TF intervention on DF-ROM and PF-ROM, isokinetic strength, balance, and jump performance in athletes with restricted DF. The results demonstrated that TF led to significant improvements in ankle ROM, DF and PF strength, as well as dynamic balance, with partial retention of these adaptations after six weeks of detraining. However, no significant enhancement in jump performance was observed. These findings highlight the potential of TF as an effective intervention for improving joint mobility and neuromuscular function, although its impact on explosive performance appears limited.

### Ankle Range of Motion

TF produced marked improvements in ankle mobility. Ankle DF ROM increased by 66.6% post-intervention versus 24.6% in the SS group, with partial retention after detraining (40.4% vs. 12.2%). PF-ROM showed smaller but still greater gains in TF (11.1% vs. 3.6%). These results indicate that TF provides superior and more durable adaptations compared to SS. Our findings are consistent with prior work reporting acute ROM gains following TF. For example, Driller and Overmayer [2] observed a 16.5% increase in DF immediately after a single application, and Konrad et al. [13] highlighted consistent short-term benefits across studies. The present results extend this evidence by demonstrating that a structured, six-week TF program yields chronic improvements that remain detectable weeks after the intervention ceases. Compared with SS, studies have shown to produce relatively modest increases in flexibility over time, TF appears to provide a more efficient mobility intervention [30, 31]. Several mechanisms may explain these outcomes. The compressive force of the floss band likely promotes fascial shearing, enhancing tissue glide and reducing myofascial restrictions. The temporary ischemia and subsequent reperfusion upon band removal may improve vascular perfusion and tissue pliability, while stimulation of cutaneous and deep mechanoreceptors may acutely reduce neuromuscular inhibition, allowing greater joint excursion [2, 13, 14, 28, 32, 33]. With repeated application across six weeks, these acute effects may have consolidated into more persistent structural and neuromuscular adaptations This likely explains the partial retention observed after detraining.

### Muscle Strength

TF produced significant improvements in ankle strength, most notably in DF but also in PF at slower testing speeds. In the dorsiflexors, CON strength at 30°/s increased by 8.5% post-intervention and remained 5.2% above baseline after detraining, while ECC strength improved by 10%, with 8.6% retained after detraining. At 120°/s, CON DF strength increased by 13.9%, with a sustained 12% gain after detraining, and ECC strength rose by 6.5%, with partial retention. At 30°/s, PF strength improved by 4% in both CON and ECC modes, with minor but sustained gains following detraining, whereas at higher velocities (120°/s), changes were minimal. The mechanisms underlying these adaptations likely extend beyond improved mobility alone. Peripherally, repeated cycles of compression and reperfusion may influence muscle and tendon stiffness, improving contractile efficiency and torque output [13]. In addition, the substantial compressive and shear forces generated during TF are thought to stimulate cutaneous and deep mechanoreceptors, enhancing motor unit recruitment and neuromuscular activation [13, 33, 34]. The persistence of strength gains after detraining suggests that TF promotes durable adaptations, which are likely neural rather than purely mechanical in nature. While previous studies have shown acute increases in force production following TF [35–37], the present results extend this evidence by demonstrating that repeated application can yield strength improvements that are sustained over several weeks without continued intervention. Despite reaching statistical significance, several strength gains were small and are unlikely to meaningfully enhance maximal athletic performance, particularly in explosive tasks. Nonetheless, even modest increases in DF strength may be clinically relevant in athletes with restricted ankle mobility by contributing to improved joint stability and neuromuscular control.

### Dynamic Balance

The YBT results demonstrated that TF elicited direction-specific improvements in dynamic balance. Posteromedial and posterolateral reach distances increased significantly post-intervention (10.3% and 7.5%, respectively), with partial retention after detraining (4% and 4.5% above baseline). In contrast, anterior reach scores showed a modest but non-significant improvement (3.5%, *p* > 0.05), suggesting that the intervention period or testing demands may have influenced outcomes. Movement variability, the natural variation in motor control strategies, may also explain the discrepancy. The study’s use of only three test repetitions may have been insufficient to account for that, potentially obscuring a true treatment effect for the anterior reach, which relies on different muscular activation patterns [38, 39]. The divergent results across YBT directions likely stem from distinct neuromuscular demands. Posteromedial and posterolateral reaches challenge the posterior chain, placing greater emphasis on ankle DF and eccentric control of the gastrocnemius-soleus complex [40]. These are areas directly targeted by TF’s compressive action during dynamic exercises like squats and lunges, which explains the significant improvements observed. In contrast, the anterior reach relies more heavily on tibialis anterior activation, a muscle group that may respond more slowly to the intervention [41, 42]. Consequently, the six-week timeframe, while sufficient for posterior-chain adaptations, might have been inadequate to induce measurable changes in the anterior direction, especially given the test’s sensitivity to subtle neuromuscular shifts. Furthermore, postural stability is integrally linked to trunk control [43, 44], and direction-specific improvements may also reflect TF’s enhanced impact on the core and posterior musculature critical for posterior-directed reaches. Longer intervention periods or higher-frequency flossing sessions could clarify whether anterior balance benefits require extended adaptation. The sustained improvements in posterior-directed balance align with TF’s proposed mechanisms: enhanced fascial glide and proprioceptive acuity [45, 46]. Maintaining balance relies on the integration of sensorimotor information [47], which is likely amplified by the band’s compression during dynamic exercises, enhancing sensory feedback to the muscle stabilizers [48] and optimizing control during reaches. This is supported by the concurrent gains in DF strength and ROM, which are critical for maintaining stability in these directions. Clinically, the findings suggest TF may be particularly valuable for athletes who prioritize lateral and diagonal movements (e.g., soccer, tennis) over purely sagittal-plane tasks. However, the non-significant anterior trend warrants caution; while TF’s primary benefits target posterior/lateral stability, its role in anterior balance remains unclear and may depend on dosage or exercise selection. Future studies could isolate anterior-chain responses by pairing TF with tibialis-focused movements or extending the intervention period to monitor delayed effects.

### Vertical Jump Performance

The SJT results revealed no significant changes in jump performance for either the TF or SS groups. In the TF group, jump height increased marginally from 43.4 ± 3.0 cm at baseline to 44.0 ± 2.9 cm post-intervention before declining to 42.9 ± 1.8 cm post-detraining, while the SS group showed slight declines from 42.4 ± 3.1 cm (baseline) to 41.9 ± 2.2 cm (post-intervention) and 41.7 ± 2.0 cm (post-detraining). This lack of improvement is consistent with the principle of training specificity. The TF protocol primarily targeted DF ROM and local neuromuscular control, which are distinct from the high-velocity, multi-joint triple extension (the simultaneous explosive extension of the ankle, knee, and hip) required for maximal vertical jumps [49]. Improvements in isolated joint function and sensorimotor control do not automatically transfer to the complex, coordinated recruitment of fast-twitch muscle fibers and the generation of high-power outputs necessary for tasks such as the SJT. Consequently, TF should not be expected to replace dedicated power-oriented interventions such as plyometrics or heavy resistance strength training. Instead, its role is likely complementary with the goal to optimize ankle mobility and stability. In this regard, TF may help to create a more effective biomechanical foundation. This could potentially improve movement quality, reduce compensatory patterns, and lower injury risk during subsequent power training, thereby allowing athletes to better express and develop their explosive capabilities through sport-specific means.

This study has several limitations that should be acknowledged. First, the sample included only young male athletes, which may limit the generalizability of the findings to female athletes, older populations, or non-athletic individuals. Second, the use of an active control group (SS) means our findings demonstrate the relative efficacy of TF compared to another common intervention, not its absolute efficacy compared to a passive control or sham condition. Third, although we measured outcomes over a six-week detraining period, longer-term follow-up is needed to determine the durability of the observed adaptations. Fourth, the intervention did not control for potential confounding factors such as variations in participants’ overall training load, nutrition, or recovery strategies, which could have influenced performance outcomes. Fifth, movement variability was not directly assessed, and the use of only three YBT trials per direction may have reduced sensitivity to detect subtle or direction-specific balance changes, especially in the anterior reach. This should be considered in future research when interpreting the balance-related results. Finally, the lack of blinding in intervention delivery may have introduced performance or observer bias. Future research with more diverse samples, a passive control group, longer follow-up, and more rigorous control of confounding variables are warranted.

## Conclusion

This study demonstrated that a six-week TF intervention significantly improved ankle ROM, strength, and dynamic balance in team- and racket-sport athletes with limited DF, with partial retention after detraining. While TF outperformed SS in terms of mobility and stability gains, it did not improve jump performance. These findings support TF as a practical, long-term strategy for enhancing ankle function in athletes, although additional training may be necessary to develop explosive power. Further research should explore optimal TF protocols and combined interventions.

## Supplementary Information

Supplementary Material 1.

Supplementary Material 2.

## Data Availability

The data that supports the findings of this study are available in the Zenodo data repository at [10.5281/zenodo.17896155].

## Abbreviations

CON: Concentric
DF: Dorsiflexion
ECC: Eccentric
ICC: Intra-Class Coefficient
PT: Plantarflexion
ROM: Range of Motion
SJT: Sargent Jump Test
SS: Static Stretching
TF: Tissue Flossing
YBT: Y-Balance Test
