# Supplementary material for: The long-term effects of tissue flossing on ankle range of motion, strength, balance, and jump performance in athletes with limited ankle dorsiflexion: a randomized controlled trial
Source: BMC Sports Sci Med Rehabil. 2026 Feb 24;18:113. doi: 10.1186/s13102-026-01609-9 (PMC12967009; doi:10.1186/s13102-026-01609-9)
Supplement: Supplementary file 1 — Supplementary Material 1. [file 13102_2026_1609_MOESM1_ESM.docx]

- To assess test–retest reliability, 15 participants were randomly selected. Measurements were taken during a familiarization session and subsequently at baseline. The consistency of the results between these two time points was analyzed to determine the stability and reliability of the measurement tools.

Supplementary file 2. Reliability and measurement error metrics

| **Variable** | **ICC (1,2)** | **SEM** | **MDC_95%_** | **CV%** |
| --- | --- | --- | --- | --- |
| DF-ROM (°) | 0.92  [0.78-0.97] | 0.27 | 0.75 | 19.74 |
| PF-ROM (°) | 0.97  [0.81-0.99] | 0.43 | 1.18 | 5.67 |
| **Isokinetic strength-30°/s** | | | |  |
| DF-CON (Nm) | 0.97  [0.92-0.99] | 2.93 | 8.12 | 28.82 |
| DF-ECC (Nm) | 0.99  [0.97-0.99] | 1.89 | 5.23 | 16.90 |
| PF-CON (Nm) | 0.99  [0.98-0.99] | 2.10 | 5.82 | 11.35 |
| PF-ECC (Nm) | 0.99  [0.98-0.99] | 6.22 | 17.24 | 26.41 |
| **Isokinetic strength-120°/s** | | | |  |
| DF-CON (Nm) | 0.98  [0.95-0.99] | 0.80 | 2.22 | 16.64 |
| DF-ECC (Nm) | 0.99  [0.97-0.99] | 1.25 | 3.46 | 16.24 |
| PF-CON (Nm) | 0.99  [0.98-0.99] | 3.43 | 9.50 | 27.62 |
| PF-ECC (Nm) | 0.99  [0.97-0.99] | 11.85 | 32.84 | 27.11 |
| **Y-balance test** | | | |  |
| YBT-ANT (%) | 0.99  [0.98-0.99] | 0.94 | 2.60 | 9.47 |
| YBT-ML (%) | 0.96  [0.88-0.9] | 0.71 | 1.97 | 5.24 |
| YBT-PL (%) | 0.99  [0.98-0.99] | 1.05 | 2.91 | 8.75 |
| Total score (%) | 0.99  [0.97-0.99] | 0.57 | 1.59 | 6.02 |
| SJT (cm) | 0.98  [0.95-0.99] | 0.29 | 0.80 | 2.43 |

**Abbreviations:** ICC, intra class coefficient; SEM, standard error mean; MDC, minimal detectable change; CV, coefficient of variation; ADF, ankle dorsiflexion; ROM, range of motion; APF, ankle plantarflexion; CON, concentric; ECC, eccentric; YBT, Y balance test; ANT, anterior; PM, posteromedial; PL, posterolateral; SJT, Sargent jump test.
