# Supplementary material for: The long-term effects of tissue flossing on ankle range of motion, strength, balance, and jump performance in athletes with limited ankle dorsiflexion: a randomized controlled trial
Source: BMC Sports Sci Med Rehabil. 2026 Feb 24;18:113. doi: 10.1186/s13102-026-01609-9 (PMC12967009; doi:10.1186/s13102-026-01609-9)
Supplement: Supplementary file 2 — Supplementary Material 2. [file 13102_2026_1609_MOESM2_ESM.docx]

**Tissue Flossing Protocol**

**Week 1-2**

- **Ankle Pumps**
  - Sets: 3
  - Duration: 60 seconds/set
  - Concentric: 1 sec (plantarflexion)
  - Eccentric: 2 sec (dorsiflexion)
  - Pause: 1 sec at end ranges
- **Bodyweight Squats**
  - Sets: 3
  - Duration: 30 seconds/set
  - Concentric: 1-2 sec
  - Eccentric: 3-4 sec
  - Pause: 1 sec at bottom
- **Weight-bearing Lunges**
  - Sets: 3
  - Duration: 45 seconds/set
  - Concentric: 2 sec
  - Eccentric: 3 sec
  - Pause: 2 sec at end ROM

**Week 3-4**

- **Ankle Pumps**
  - Sets: 3
  - Duration: 75 seconds/set
  - Concentric: 1 sec
  - Eccentric: 2-3 sec
  - Pause: 1 sec at end ranges
- **Bodyweight Squats (Introduce Light Load if ready)**
  - Sets: 3
  - Duration: 40 seconds/set
  - External Load: Optional light dumbbells
  - Concentric: 1-2 sec
  - Eccentric: 3-4 sec
  - Pause: 1 sec at bottom
- **Weighted Lunges (Light Load)**
  - Sets: 3
  - Duration: 60 seconds/set
  - Concentric: 2 sec
  - Eccentric: 3 sec
  - Pause: 2 sec at end ROM

**Week 5-6**

- **Ankle Pumps with Band Tension**
  - Sets: 3
  - Duration: 75 seconds/set
  - Concentric: 1 sec
  - Eccentric: 2-3 sec
  - Pause: 1 sec at end ranges
- **Loaded Bodyweight Squats**
  - Sets: 3
  - Duration: 45 seconds/set
  - External Load: Increase based on ability
  - Concentric: 1-2 sec
  - Eccentric: 3-4 sec
  - Pause: 1 sec at bottom
- **Weighted Lunges (Progressive Load)**
  - Sets: 3
  - Duration: 60 seconds/set
  - Load: Increase based on ability
  - Concentric: 2 sec
  - Eccentric: 3 sec
  - Pause: 2 sec at end ROM

**Passive Static Stretching Protocol**

- **Week 1-6**
- **Gastrocnemius Stretch**
- Stretch Type: Standing Wall Stretch (straight back leg, knee extended)
- Sets: 3
- Duration: 30 seconds per set
- Rest: 15 seconds between sets
- **Soleus Stretch**
- Stretch Type: Standing Wall Stretch (back leg bent, knee slightly flexed)
- Sets: 3
- Duration: 30 seconds per set
- Rest: 15 seconds between sets
